# Transformer-based land use and land cover classification with explainability using satellite imagery

**DOI:** 10.1038/s41598-024-67186-4

**Published:** 2024-07-20

**Authors:** Mehak Khan, Abdul Hanan, Meruyert Kenzhebay, Michele Gazzea, Reza Arghandeh

**Affiliations:** Department of Computer Science, Electrical Engineering and Mathematical Sciences, Western Norway University of Applied Sciences, Bergen, Norway

**Keywords:** Computer science, Ecology

## Abstract

Transformer-based models have greatly improved Land Use and Land Cover (LULC) applications. Their revolutionary ability to analyze and extract key information has greatly advanced the field. However, the high computational cost of these models presents a considerable obstacle to their practical implementation. Therefore, this study aims to strike a balance between computational cost and accuracy when employing transformer-based models for LULC analysis. We exploit transfer learning and fine-tuning strategies to optimize the resource utilization of transformer-based models. Furthermore, transparency is the core principle of our methodology to promote fairness and trust in applying LULC models across various domains, including forestry, environmental studies, and urban or rural planning. To ensure transparency, we have employed Captum, which enables us to uncover and mitigate potential biases and interpret AI-driven decisions. Our results indicate that transfer learning can potentially improve transformer-based models in satellite image classification, and strategic fine-tuning can maintain efficiency with minimal accuracy trade-offs. This research highlights the potential of Explainable AI (XAI) in Transformer-based models for achieving more efficient and transparent LULC analysis, thereby encouraging continued innovation in the field.

## Introduction

Investigating Land Use and Land Cover (LULC) is crucial for understanding the complex relationship between human activities and the environment. It highlights how we use land vital for diverse applications, including forestry, precision agriculture, urban or rural planning, environmental protection, disaster response, and sustainable practices. Studying changes in land use and land cover aids in efficient land management strategies for policy-makers and academicians^1,2^.

Deep learning models, particularly Transformers, have been a cornerstone in computer vision and significantly improved the ability of LULC applications^3,4^. The extraordinary precision of these models has transformed how different land cover and types can be analyzed and distinguished. Recent developments in transformer-based models for image classification have proven that the Vision Transformer (ViT) model provides several advantages over traditional Convolutional Neural Network (CNN) models. Initially introduced by Vaswani et al.^5^, this transformer model delivered impressive performance in Natural Language Processing (NLP) tasks. As a result, researchers have adapted this transformer model to various computer vision tasks due to its success in NLP^6–11^.

Transformers have shown great potential in various remote sensing tasks beyond Image classification, including segmentation and object detection. Their self-attention mechanism enables them to model long-range dependencies and interactions within images, making them highly effective for complex applications. For example, in instance segmentation, transformers can accurately segment individual objects within a scene by capturing contextual information across the image. In object detection, models like DETR (DEtection TRansformers)^12^ have transformed the field by processing entire images in a single pass, improving detection efficiency and accuracy. These capabilities expand the usefulness of transformers beyond traditional image classification to more specialized and demanding tasks in remote sensing.

Unlike traditional CNNs, which primarily focus on identifying image features without including positional relationships among these features, thus lacking a comprehensive understanding of the entire image, the ViT model proposed by Dosovitskiy et al.^6^ proposes a solution. By utilizing self-attention layers, the ViT model can learn images globally and reduce the image-specific inductive bias, thereby addressing the limitations of traditional CNN approaches.

Touvron et al.^13^ introduced the Data-efficient image Transformers (DeiT) to reduce computational costs and enhance the efficiency of the transformer-based models in image classification tasks. Despite advancements in new transformer models like ViT and DeiT, processing images of various domains and scales remains challenging. To address this, Liu et al.^14^ introduced the Swin Transformer (SwinT) model, a breakthrough in handling different image scales. This model is proficient at adjusting its processing to efficiently identify objects at various scales, demonstrating exceptional performance in image classification tasks. Its ability to maintain high accuracy across diverse image types and sizes makes it a significant advancement in the field, especially for applications in image classification. However, applying these Transformer-based models in remote sensing for image classification and analysis has received relatively slight attention. To fill this gap, Jannat et al.^15^ explored the use of these transformer-based models for classifying LULC in satellite imagery. The key contributions of this work include demonstrating that the SwinT model outperforms ViTs in accurately classifying remote sensing images on benchmark datasets, including EuroSAT. This research plays a pivotal role in enhancing the performance of advanced transformer-based models for LULC classification. However, it does not specifically address the computational costs associated with these improvements.

This is a crucial aspect to consider, as achieving such high accuracy often requires substantial computational resources, posing a major challenge in the field^16^. Consequently, a key question in the evolution of computer vision is finding a balance between the accuracy of these models and the computational costs needed to produce reliable results. This challenge highlights the need for further research and development to optimize the efficiency of advanced transformer-based models.

Thus, in this paper, we utilize transfer learning and fine-tuning, which have opened new avenues for mitigating the problem of high computation costs that often come with transformer-based models. These techniques leverage pre-trained models trained on extensive datasets, which help reduce the computational cost while sustaining the level of accuracy. In contrast, fine-tuning strategically utilizes layers in models to optimize their performance. These strategies make complex computer vision models more accessible by enhancing their efficiency. By leveraging these models, we can maintain a balance between computation cost and precision, which offers a promising direction for research in computer vision and LULC classification^17^.

In addition to the challenges of balancing computational cost and accuracy in advanced deep learning models for LULC classification, there is an issue associated with the complex and black-box nature of these advanced deep learning models. Therefore, making them transparent is becoming increasingly essential to understand how decisions are made. Although these models are highly accurate, their inner workings lack explanations, making it challenging to understand their decision-making process. To address this transparency issue, Explainable AI (XAI) can be used to make deep learning models transparent to enrich their clarity for humans^18^.

In the context of remote sensing data, various XAI techniques have been applied to analyze satellite imagery, particularly for LULC classification tasks. Some notable methods include Grad-CAM^19^, local interpretable model-agnostic explanations (LIME)^20^, Shapley additive explanation (SHAP)^21^. However, there are significant constraints to applying these XAI techniques to transformer-based models for LULC classification. Due to their complexity, these XAI techniques might not fully comprehend the transformer-based model’s decision-making processes, which could limit interpretability. Accordingly, Captum, introduced by Kokhlikyan et al.^22^, has been suggested as a valuable addition for understanding the decisions made by transformer-based models in LULC classification. The Captum tool is designed to be independent of any particular neural network architecture, allowing easy integration without requiring the original network. This facilitates their use and implementation in transformer-based LULC classification models, removing the need for significant adjustments. By providing insights into what drives their outputs, such tools play a vital role in ensuring the reliability and trustworthiness of the models in LULC analysis. Their use is a significant step towards making advanced computer vision models more interpretable and accountable, which is essential for their broader acceptance and application related to LULC classification.

By integrating Captum into our LULC framework, we aim to achieve two objectives. Firstly, it can promote trust, fairness, and clarity, which is essential for practitioners who depend on LULC AI-driven decisions for critical environmental, rural, or urban planning decisions. Secondly, it helps refine the model by alleviating light on the reason behind predictions made by the model. Researchers can benefit from such information to identify and correct biases in data, thus optimizing the performance and reliability of the models.

Therefore, this paper introduces a framework specifically designed to enhance the efficiency of transformer-based models by employing transfer learning and fine-tuning strategies. It also examines the significance of interpretability in these models, with a particular emphasis on LULC analysis. The goal is not only to improve the performance of these advanced deep-learning models but also to ensure that their decision-making processes are transparent and understandable. This is a critical consideration for practical applications in LULC classification, including forestry, precision agriculture, urban or rural planning, environmental protection, disaster response, and sustainable practices.

The main contributions of this paper are summarized as follows:

- This study aims to improve the efficiency of Vision Transformer and Swin Transformer models through transfer learning and fine-tuning.
- Explores the significance of making advanced deep learning models more understandable and transparent by highlighting the importance of fairness, transparency, and trust for practical and ethical use in LULC analysis.
- Experimental results demonstrate that transfer learning can significantly improve the performance of transformer-based models for satellite image classification and LULC analysis. Concurrently, strategic fine-tuning can reduce computational costs with only a minimal drop in accuracy.

**Figure 1:**
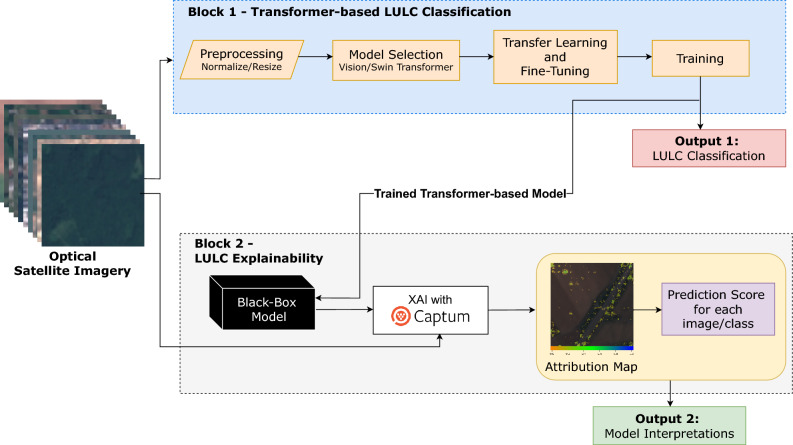
Overview of the proposed framework for transformer-based LULC classification with explainability using satellite imagery.

## Methodology

This study proposes an innovative framework that uses optical satellite imagery for LULC classification. This framework distinguishes itself by exploiting the advanced capabilities of transformer-based models. A notable aspect of this framework is its focus on explainability, integrated into its algorithmic architecture. This integration of explainability ensures that the framework not only performs its task effectively but also provides insights into the decision-making process of the black-box transformer-based model, making it a robust tool in the field of satellite-based LULC analysis. The proposed framework is divided into two main blocks, Transformer-based LULC and Explainability, as depicted in Fig. 1.

### Transformer-based LULC classification

The Transformer-based LULC Classification is the *first block* in our proposed framework that uses transformer-based models for LULC classification. This block begins with a crucial preprocessing step. In this step, satellite images are meticulously transformed to align with the specific requirements of transformer-based models. This involves resizing the images to a uniform dimension to ensure consistency across the entire dataset. Moreover, the images undergo a normalization process of their color channels - Red, Green, and Blue. This involves adjusting each channel based on a calculated mean and standard deviation. These steps of resizing and normalization are vital. They ensure that the data is uniformly structured, which is essential for consistent and reliable analysis and interpretations by the model.

**Figure 2:**
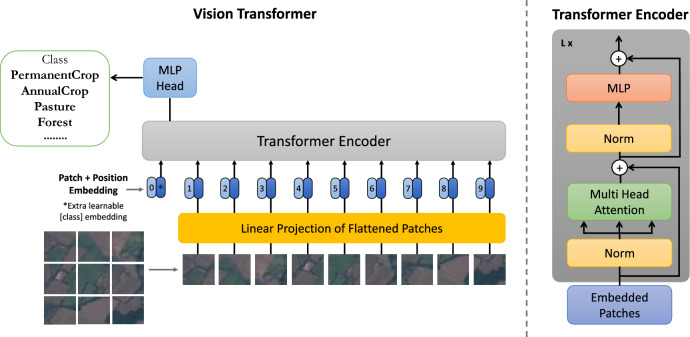
Architecture of vision transformer (ViT)^6^.

As part of our model selection process, we mainly focused on two models: *Vision Transformer (ViT)* and *Swin Transformer (SwinT)*. We chose these models due to their effective attention mechanisms. However, it is integral to note that this framework can also use other deep learning models as baselines for LULC analysis.

#### Vision transformer (ViT)

We utilized the ViT model for LULC classification of satellite imagery, which was initially designed for image classification^6^. Our approach includes two versions of the ViT model: *ViT-Base* and *ViT-Large*, each with unique attributes to address different aspects of LULC classification, The architecture representation of ViT is illustrated in Fig. 2.

Both the ViT-Base and ViT-Large variants of the ViT use a consistent method to analyze satellite images. It starts with resizing the images to a uniform $224 \times 224$ pixel resolution. Then, they are divided into smaller $16 \times 16$ pixel patches and flattened. This transformation converts the original two-dimensional image array into a sequence of one-dimensional vector embeddings, similar to the tokenization process used in natural language processing. To address the transformer architecture’s lack of inherent positional understanding, positional embeddings are added to these embeddings. Including positional information is crucial for the model to interpret the spatial dynamics of the satellite imagery accurately.

After generating the embeddings, the transformer encoder processes a sequence of these embeddings through a series of operations. One of these operations is the Multi-Head Attention mechanism, which calculates attention scores to enable focusing on different image segments. This equation quantifies the process:

1 $$\begin{aligned} \text {Attention}(Q, K, V) = \text {softmax}\left( \frac{QK^T}{\sqrt{d_k}}\right) V \end{aligned}$$

where *Q* represents the query matrix, *K* signifies the key matrix, and *V* denotes the value matrix. The $QK^T$ represents the matrix multiplication of *Q* with the transpose of the *K* to calculate the attention scores, and the dimensionality of the keys and queries indicated by $d_k$ acts as the scaling factor for the softmax calculation.

After the attention layer, the network’s output is stabilized by a normalization step. To prevent overfitting, Dropout is used as a regularization technique. The Multi-layer Perceptron (MLP) block transforms the data through fully connected layers using GELU (Gaussian Error Linear Units) as the activation function, providing non-linearity^23^:

2 $$\begin{aligned} \text {GELU}(x) = x \cdot \Phi (x) \end{aligned}$$

where $\Phi (x)$ represents the cumulative distribution of the Gaussian distribution.

After completing these steps, the transformer encoder outputs are directed to a MLP head, which functions as the classifier. It converts the acquired representations to the dimensionality of the LULC categories, including ’PermanentCrop,’ ’AnnualCrop,’ ’Pasture,’ ’Forest’, and so on. Once again, the dropout layer is applied to ensure model generalization before the final linear classification layer.

The ViT-Large model has approximately 304 million parameters, making it ideal for complex pattern recognition tasks due to its additional layers and higher capacity. It is best suited for tasks that require detailed feature interactions. On the other hand, the ViT-Base model has around 86 million parameters and is optimized for efficiency, making it outstanding for less demanding tasks that require faster processing times. This signifies the adaptability of the ViT model in handling complex tasks like LULC classification. It can easily transition from image preprocessing to final classification with robustness and effectiveness.

**Figure 3:**
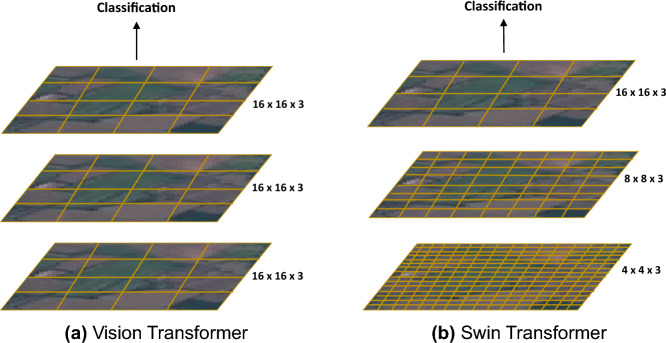
Vision transformer vs swin transformer.

#### Swin transformer (SwinT)

We incorporated SwinTs alongside ViTs in our framework for their distinctive approaches to handling satellite imagery for LULC classification. It was initially proposed for image classification with a distinctive approach to improve ViTs^14^. ViTs segment images into a grid of non-overlapping patches and treat each patch equally in the global self-attention process. This approach is ideal for aggregating contextual information across the entire image but can be computationally expensive and may miss localized features. On the other hand, SwinTs use shifted windows and a hierarchical design to focus on subsets of these patches, as illustrated in Fig. 3. This window-based mechanism allows SwinTs to capture fine-grained details with greater computational efficiency. It makes them ideal for processing high-resolution imagery where precise local feature delineation is critical. The choice of which model to use depends on the specific requirements of the analysis task: ViTs for a broader, global perspective and SwinTs for a more detailed attention to local spatial features.

Our proposed framework utilizes the SwinT architecture to effectively process an input image with dimensions $H \times W \times 3$. The process is initiated by dividing the image into $16 \times 16$ pixel patches. These patches are then transformed into 256-dimensional feature vectors through a Linear Embedding layer, yielding the initial feature set $z^0$. As the model progresses through its stages, the feature representations evolve from $z^0$ to $z^l$ and subsequently to $z^{l+1}$. Each stage includes Patch Merging operations that not only reduce the spatial dimensions but also double the number of channels *C*, thus deepening the feature representation to dimensions such as $\frac{H}{32} \times \frac{W}{32} \times 2C$ and $\frac{H}{64} \times \frac{W}{64} \times 4C$, enhancing the model’s ability to analyze and classify various land covers.

**Figure 4:**
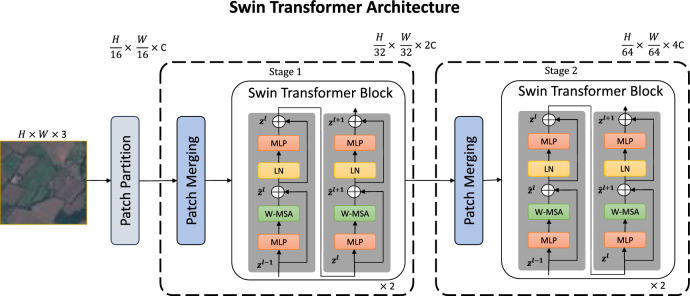
Architecture of swin transformer^14^.

Within each SwinT Block, the Window-based Multi-head Self-attention (W-MSA) and Shifted Window Multi-Head Self-attention (SW-MSA) mechanisms are pivotal. W-MSA focuses on extracting localized features within each window, whereas SW-MSA shifts the window to capture extended contextual information, ensuring comprehensive coverage for LULC classification. Layer Normalization (LN) is systematically applied before each self-attention module to facilitate stable training and normalize feature distributions. Following attention processing, the MLP blocks perform channel-wise feature transformations, transitioning the representations from $z^l$ to $z^{l+1}$. Within the MLP, GELU, and dropout are employed to support the transformation process. This step is critical for the model to decode complex and non-linear patterns associated with different LULC categories. The SwinT architecture is visually depicted in Fig. 4. The SwinT architecture is proficient at LULC classification, showcasing an enhanced capability to distinguish fine spatial details across broad landscapes, including urban/rural developments and natural environments. As a tool for environmental monitoring, resource management, and land change detection, the model provides precise and actionable insights, supporting the sustainable management of land resources. With around 49 million trainable parameters, this indicates its advanced complexity and remarkable learning ability. Its comprehensive layer configuration and extensive parameter count make it an excellent tool for handling complex challenges in satellite image analysis for LULC classification.

#### Transfer learning and fine-tuning

To improve the effectiveness of transformer-based models, we employ transfer learning and fine-tuning techniques in our framework. Transfer learning allows us to utilize the knowledge gained from pre-trained models, significantly reducing the high computational challenges usually associated with these models^24^. This approach not only improves accuracy but also makes these advanced models more accessible for various uses. The fine-tuning process is equally crucial, and it involves detailed, layer-wise adjustments that are carefully adapted, mainly focusing on selectively unfreezing and training the last few layers of these models^25^. In the fine-tuning process, we first freeze all the initial layers of the model to preserve the generic features they have learned, as these layers are critical for capturing fundamental, broadly applicable features. Then, we shift our focus to the last few layers, especially the last two/three blocks, selectively unfreezing and training them. This step is essential since these layers are more specialized and tasked with learning higher-order, task-specific features. By concentrating our fine-tuning attempts on these layers, we efficiently adapt the model to the specific requirements of the task at hand. This strategy of freezing the initial layers and selectively unfreezing the later ones is an effective way to fine-tune models. It retains the foundational knowledge captured by the initial layers while refining the more advanced features in the later layers. This approach not only strengthens the model’s performance for specific tasks but also significantly cuts down on computational costs and training time, as opposed to retraining the entire model from scratch. The combination of transfer learning and fine-tuning enhances the accuracy, transparency, and interpretability of the models, ensuring compliance with the highest ethical standards in remote sensing. By harmonizing these two techniques, our framework significantly contributes to emphasizing the significance of responsibly and sustainably advancing LULC analysis applications.

#### Training

After integrating these discussed transformer-based models, transfer learning, and fine-tuning techniques, our first block moves on to a rigorous training phase using the settings explained in section “Training settings”, wherein it learns to decode the intricate patterns in satellite imagery. LULC classification results are obtained through this process, which comprehensively analyzes various land cover types such as forests, agricultural lands, and urban/rural areas. This information is incredibly valuable for various LULC applications such as environmental analysis and land use planning purposes.

**Table 1:** Comparison of XAI methods for transformer models in vision.

XAI factors	Captum^22^	Grad-CAM^19^	SHAP^21^	LIME^20^
Focus on predictions	Offers high-resolution, instance-specific analysis by attributing output prediction scores to each input feature.	Provides a localization map highlighting regions relevant for a particular class, with less resolution.	Delivers feature-level attributions but may not delve into specific complexities of transformers.	Uses a linear surrogate, which is less precise for complex models like transformers.
Layer-specific insights	Gives detailed insights at the layer level, valuable for transformers’ complex architectures.	Limited to convolutional layers, offering less detail for transformer architectures. Not optimized for transformers.	Provides feature-level insights, not specifically focused on layer-specific dynamics.	Does not offer detailed insights on layer-specific contributions.
Model specificity	Tailored for deep learning and transformer models, adept at capturing intricate interactions.	Mainly designed for CNNs, not optimized for the specific structure of transformers.	Versatile across models but may not capture the full depth of transformer intricacies.	Less specific for complex models like transformers due to its generalist approach.
Computational efficiency	Efficient for complex models like transformers optimized for deep learning structures.	Efficient but not fully adapted to leverage transformer structures.	Resource-intensive for large models like transformers.	Efficient but may struggle with the complexity of transformers.
Attribution accuracy	Provides highly accurate and consistent attributions for each input feature.	Good at highlighting important regions but less precise for detailed feature-level attribution.	Offers a balance of accuracy but less focus on transformer-specific complexities.	Less accurate for complex dependencies typical in transformer models.
Handling complex data structures	Effectively capture complex data structures by working directly with the original model.	Focuses on spatial relevance, which may not fully address complex data relationships in transformers.	Capable of handling complex structures but might not capture all nuances of transformers.	Less effective in capturing the complex structures and dependencies typical in transformer models.

### Explainability

The Captum tool provides a user-friendly way to interpret machine learning models, especially when dealing with different kinds of data (multi-modal), like images and text. It works well with numerous PyTorch models and doesn’t require significant changes to the original neural network. Additionally, it’s an open-source library that’s handy for anyone looking to delve into how AI interprets information, making it more straightforward to try and test new methods and ideas. To provide a better understanding, we have compared Captum with other notable XAI methods in the context of transformer-based models in Table 1.

In the second block of our proposed framework, we have integrated the Captum library’s Integrated Gradients tool, a significant advancement in the field of XAI. This tool is employed to interpret intricate satellite imagery analyzed by transformer-based models, traditionally seen as ’black-box’ models. The Integrated Gradients tool reveals the internal workings of these models by generating attribution maps, which are invaluable for offering deep insights into the model’s decision-making processes. These maps do this by highlighting crucial features within the imagery and assigning prediction scores for each class, thus quantifying the model’s predictive confidence.

This equation defines the functionality of the Integrated Gradients tool^22,26^:

3 $$\begin{aligned} \text {IntegratedGradients}_i(\textbf{x}):= (x_i - x'_i) \cdot \int _{\alpha =0}^{1} \frac{\partial F(x' + \alpha \cdot (\textbf{x} - x'))}{\partial x_i} \, d\alpha \end{aligned}$$

where $\text {IntegratedGrads}_i(\varvec{x})$ represents the attribution of the $i$-th feature of input $\varvec{x}$, $x_i$ is the value of the $i$-th feature, $x'_i$ is the baseline value of the $i$-th feature, $\alpha$ is a scalar that scales the input from the baseline $x'$ to the actual input $\varvec{x}$, $F$ is the model function, and $\frac{\partial }{\partial x_i}$ is the partial derivative with respect to the $i$-th feature. The integral calculates the accumulation of the gradients as the input scales from the baseline to the original value.

The integration of the Captum library into our framework marks a significant enhancement in the transparency of complex transformer-based models, a change that is critically important in sectors like environmental assessment and urban/rural development. A transformation from black-box to transparent approaches is essential for ensuring clarity and precision in AI-driven decisions. A key attribute of the Captum library is its capacity to create attribution maps, which provide invaluable qualitative insights into the AI’s decision-making process. This boosts confidence in the model’s outcomes and sharpens their accuracy. Notably, these maps contribute to identifying and rectifying potential biases within the data, leading to a more equitable and trustworthy AI system. In the context of high-stakes LULC applications, with their wide-ranging implications, the transparency provided by these tools is crucial in verifying the reliability and maintaining the model’s integrity.

**Figure 5:**
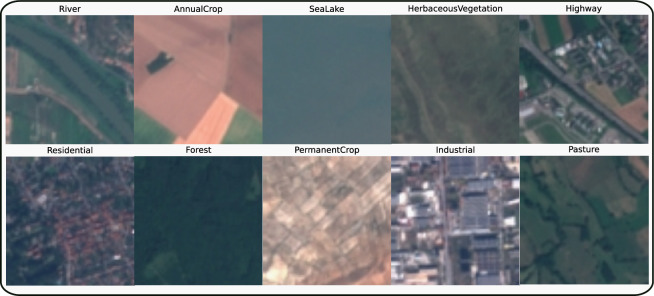
Sample images from each class in the EuroSAT dataset^27^.

**Table 2:** Dataset description.

Dataset	Type	Resolution	Image size	Bands	No. of images	Classes	Train/test size
EuroSAT-RGB^27^$$^{1}$$	Sentinel-2	10 m	64 × 64	3	27,000	10	21,600/5400

$^{1}$Data available at https://github.com/phelber/EuroSAT.

**Table 3:** Comprehensive overview of evaluation metrics for LULC analysis.

Metric	Purpose of using metric	Formula
Accuracy	Measures the proportion of correct predictions.	$\frac{\text {Number of Correct Predictions}}{\text {Total Number of Predictions}}$
Cross-entropy loss	Assesses mistakes made by the model.	$-x[c] + \log (\sum \exp (x[j]))$
Precision	Evaluates accuracy of positive predictions.	$\frac{\text {True Positive}}{\text {True Positive} + \text {False Positive}}$
Recall	Measures the ability to capture relevant instances.	$\frac{\text {True Positive}}{\text {True Positive} + \text {False Negative}}$
F1-score	Harmonizes precision and recall into a single metric.	$2 \times \frac{\text {Precision} \times \text {Recall}}{\text {Precision} + \text {Recall}}$
Balanced accuracy	Ensures equal contribution of each class, especially in imbalanced datasets.	$\frac{1}{2} \left( \frac{\text {True Positive}}{\text {True Positive} + \text {False Negative}} + \frac{\text {True Negative}}{\text {True Negative} + \text {False Positive}} \right)$
Confusion matrix	Visual comparison of predictions versus actual outcomes.	–
Computation time	Model efficiency in terms of training time required to reach convergence.	–
t-SNE	Provides valuable insights into the performance of models when navigating complex data patterns.	–

## Experimental settings

In this section, we provide a thorough overview of the dataset, training settings, evaluation metrics, and comparative analyses used in our study. This detailed description is intended to improve the clarity and readability of our study in the context of LULC classification.

### Dataset

To evaluate the performance in this study, we utilize the EuroSAT dataset^27^. The EuroSAT dataset contains Sentinel-2 satellite imagery covering three spectral bands (Red, Green, and Blue), with 27,000 labeled images and ten LULC classes, as illustrated in Fig. 5 and Table 2.

**Figure 6:**
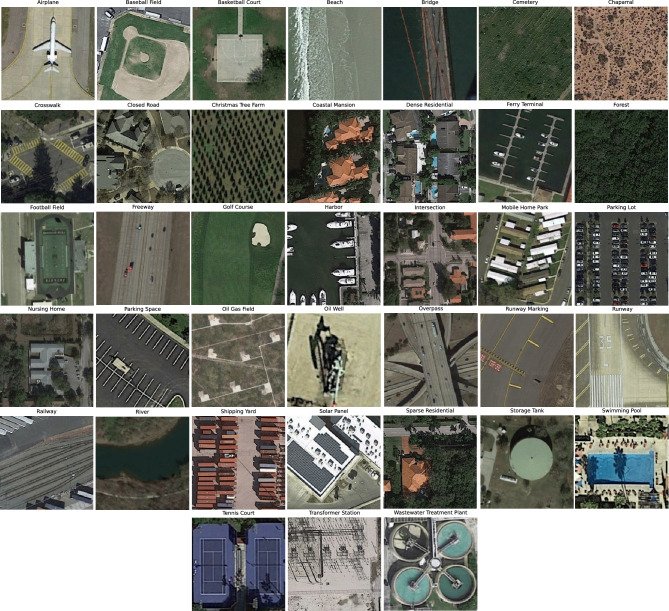
Sample images from each class in the PatternNet dataset^28^.

For further validation of our framework’s generalization and scalability, we conducted additional experiments using the PatternNet dataset^28^. PatternNet is a high-resolution remote sensing dataset designed for LULC classification. It consists of high-resolution aerial images with a spatial resolution of approximately 0.3 meters and 38 classes with 800 images per class, covering diverse urban and suburban areas. Some sample images from each class of this dataset are illustrated in Fig. 6.

### Training settings

Most of the models used in this study are from the timm library^29^, pre-trained on ImageNet-21k, a dataset consisting of 14 million images and 21k classes. For experiments, we used Google Colab’s Pro+ A100 GPU and focused on optimizing the performance of machine learning models. We implemented an early stopping mechanism, setting a threshold at 5. This revealed an intriguing pattern: most models plateaued in learning efficiency, typically reaching convergence around the 10th epoch. This suggested an optimal balance between learning depth and computational efficiency. We employed the Stochastic Gradient Descent (SGD) optimizer to enhance our model’s performance, with a learning rate set at 0.001. Our experimentation included testing various batch sizes, specifically 8, 16, 32, 64, and 128, to determine the most effective configuration. It was observed that the models performed best with a batch size of 16. Additionally, we utilized cross-entropy loss as our loss criterion, which proved effective in our study.

### Evaluation metrics

In our investigation to rigorously assess the efficacy of machine learning models, we’ve adopted a holistic approach by utilizing a diverse range of metrics. Each of these metrics is pivotal in determining a comprehensive understanding of a model’s capabilities. Table 3 provides the list of used matrices with a description and purpose of use.

### Comparative analysis

In order to compare the performance of the transformer-based LULC block in our proposed framework, we consider several deep learning architectures, including: 1) ResNet50^30^; 2) ResNet101^30^; 3) Inception V3^31^; 4) DenseNet161^32^; 5) GoogleNet^33^; 6) Vision Transformer-Base (ViT-Base)^6^; 7) Vision Transformer-Large (ViT-Large)^6^; 8) Data-efficient image Transformers-Base (DeiT-Base)^13^; 9) Swin Transformer-Small (SwinT-Small)^14^ and 10) Swin Transformer-Large (SwinT-Large)^14^.

## Results and analysis

In this section, we will present the results obtained from our extensive experiments and the subsequent analysis.

### Transformer-based LULC classification

To evaluate the performance of the transformer-based LULC block in our proposed framework, we compare it against a variety of deep learning architectures applied to the EuroSAT dataset, including several models from both the CNN and transformer families listed in section “Comparative analysis”.

#### Performance comparison of transformer-based models with other baselines on the EuroSAT dataset

Table 4 comprehensively analyzes different deep learning models on the EuroSAT dataset, assessing their performance through various crucial metrics. All of the models presented are trained using transfer learning.

ViT-Large is particularly noteworthy among these models, excelling in test loss and test accuracy. Its low test loss implies remarkable precision in predictions, while its high test accuracy indicates a powerful capability in correctly classifying images. These features underline the advanced predictive and classification ability of the ViT-Large model, making it an optimal choice for applications where accuracy is of utmost importance.

**Table 4:** Performance comparison of of non-transformer-based baselines and transformer-based models on the EuroSAT dataset.

Models	Test loss	Test accuracy	F1-score	Balanced accuracy	Computation time (min)	Parameters	Epochs
ResNet50	0.1111	96.57	96.57	96.45	32.3	23,528,522	20
ResNet101	0.0875	97.22	97.22	97.17	62.4	42,520,650	22
InceptionV3	0.1137	96.63	96.62	96.49	**24.9**	25,132,754	10
DenseNet161	0.0715	97.69	97.69	97.60	49.5	28,681,000	18
GoogLeNet	0.1906	94.22	94.23	94.02	34.8	**6,624,904**	27
SwinT-Small	0.0516	98.28	98.28	98.23	48.8	49,728,418	10
SwinT-Large	0.0586	98.20	98.20	98.10	76.7	228,565,093	10
ViT-Base	0.0399	98.70	98.71	98.66	31.6	86,5676,56	10
ViT-Large	**0.0290**	**99.11**	**99.11**	**99.07**	111.0	304,326,632	10
DeiT-Base	0.0467	98.50	98.51	98.43	34.2	86,567,656	10

Significant values are in bold.

An essential highlight of the analysis in Table 4 is the effectiveness of transformer-based models, especially ViT-Large and ViT-Base, in addressing class imbalances, a prevalent issue in machine learning where certain classes are less represented in the training data, often leading to model bias. These models demonstrate their adeptness at overcoming this challenge, as shown by their high f1-scores and balanced accuracy. The f1-scores are crucial in imbalanced datasets as they are a harmonic mean of precision and recall, ensuring minimal misclassification of false positives and false negatives. Balanced accuracy further complements this by modifying the accuracy metric to represent each class equally, providing a more equitable measure of model performance. The impressive results of ViT-Large and ViT-Base in handling class imbalances, as reflected in these metrics, signify their appropriateness for datasets with unequal class distributions, making them valuable choices in such scenarios. While not as highlighted in the analysis, the SwinT-Small, SwinT-Large, and DeiT-Base remain key players due to their distinctive architecture. Although not the most prominent compared to similar models, their effectiveness in handling class imbalances still marks it as a competent choice for such challenges. This aspect showcases the versatility of transformer-based models, such as the SwinTs, in adeptly managing datasets with varied class distributions, emphasizing their broad applicability in diverse machine-learning scenarios.

InceptionV3 sets itself apart from the more resource-heavy transformer models through its computational efficiency. It offers reliable accuracy with less resource usage and is ideal for fast model training and deployment in scenarios with constrained resources, like real-time analysis applications. Alongside this, the varying complexity of models, as indicated by their parameters, is noteworthy. GoogLeNet, with fewer parameters, presents a more straightforward option that is advantageous in settings with limited hardware systems or where straightforward interpretation and debugging are essential, catering to specific operational requirements.

Based on the insights from Table 4, it becomes evident that the number of training epochs plays a vital role in fine-tuning model performance on the EuroSAT dataset. The careful calibration of these epochs is crucial to circumvent underfitting and overfitting. Models such as ViT-Large and InceptionV3 symbolize this principle by incorporating convergence strategies like early stopping, thus optimizing their training for accuracy and adaptability. This strategic modulation of training epochs is a key factor in achieving strong and consistent performance, both on the training data and in new, unencountered scenarios, highlighting a refined approach to model training in machine learning.

This detailed evaluation offers valuable insights into the capabilities of each model, guiding the selection of the most suitable one for LULC analysis based on accuracy and other key performance indicators.

**Table 5:**
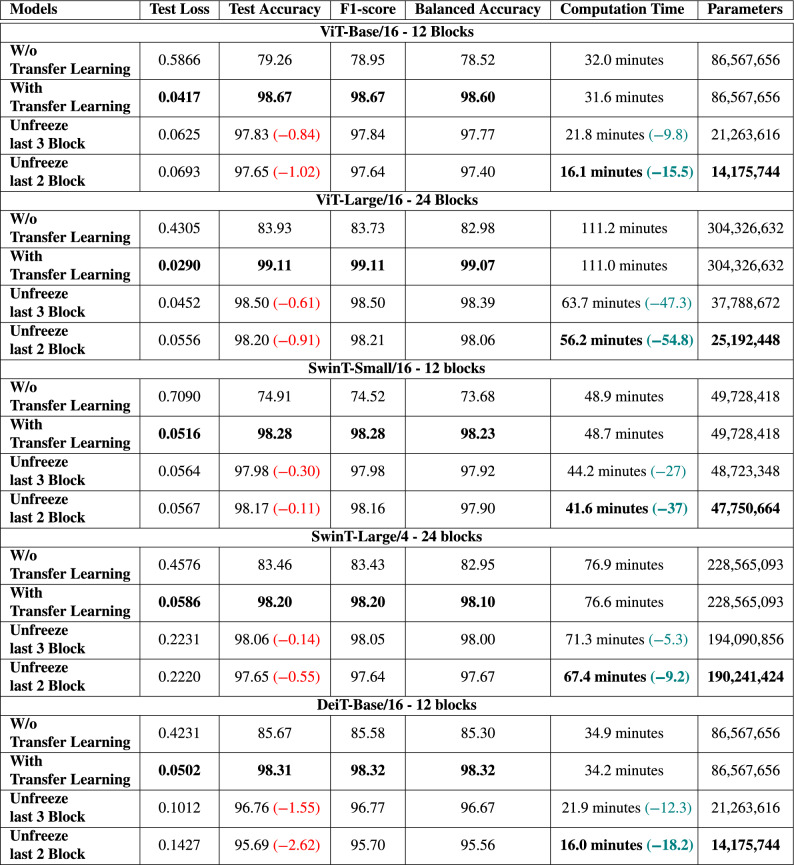
Performance comparison among transformer-based models utilizing transfer learning and fine-tuning on the EuroSAT dataset.

Significant values are in bold.

#### Transfer learning and fine-tuning over transformer-based models on EuroSAT dataset

Table 5 provides a detailed evaluation of various transformer-based architectures on the EuroSAT dataset. It compares the performance of models with and without transfer learning, as well as under different fine-tuning strategies that involve freezing specific blocks. The table compares key metrics such as test loss, test accuracy, f1-score, balanced accuracy, computation time, and the number of trainable parameters. With this comparison, we can assess how transfer learning and fine-tuning each block affect the models’ overall effectiveness and efficiency.

The ViT-Base model comprising of 12 blocks shows a significant performance improvement when transfer learning is applied. This is evidenced by a reduction in test loss and an enhancement in accuracy and f1-score, with a slight reduction in computation time. By unfreezing the last three blocks and fine-tuning, there is a marginal decrease in test accuracy but a significant improvement in computational efficiency and a reduction in the number of parameters. Further efficiency can be achieved by unfreezing the last two blocks but with a small additional compromise in accuracy. Hence, the model demonstrates a clear trade-off between accuracy and efficiency gains through strategic fine-tuning.

Transfer learning has been proven to be highly effective when applied to the ViT-Large model with 24 blocks. This technique improves the model’s test accuracy and f1-score, resulting in a score of over 99% while achieving high Balanced Accuracy. By unfreezing the last three blocks and fine-tuning the model further, the performance metrics, such as test accuracy and f1-score, show a slight decline, which is marginal and typically considered acceptable in practice. However, this fine-tuning strategy offers significant computational benefits, reducing computation time by almost 50% and reducing the model’s complexity, as indicated by the reduced number of parameters. This efficiency gain is beneficial for practical applications where resource constraints are a concern. If only the last two blocks are unfrozen during the fine-tuning process, the performance metrics show a similar but smaller decrease, contributing to reduced computation time and fewer parameters. This suggests a diminishing return on efficiency gains with fewer blocks unfrozen, but it highlights the customizable nature of the fine-tuning process to balance between model performance and computational efficiency.

The SwinT-Small model with 12 blocks has significantly improved all key performance indicators when subjected to transfer learning. This model is designed with a smaller architecture, which makes it possible to transfer knowledge from pre-trained networks, resulting in improved test loss, test accuracy, f1-score, and balanced accuracy. Moreover, transfer learning reduces computation time, thereby enhancing computational efficiency for this model. When fine-tuning strategies are implemented by unfreezing the last three blocks of the model, there is a slight drop in test accuracy and balanced accuracy. However, this trade-off is minimal, and the benefits of computational efficiency are evident as there is a reduction in computation time. Fine-tuning helps the model reach convergence faster, which can be particularly beneficial when limited resources or rapid model deployment is necessary. Further fine-tuning, which involves unfreezing the last two blocks of the SwinT-Small, results in largely consistent performance metrics with the previous configuration, maintaining a similar level of accuracy. However, this approach yields additional reductions in computation time, making it a more cost-effective option in cases where time and computational power are limited.

The SwinT-Large model with 24 blocks significantly benefits from transfer learning, demonstrating an improvement in test loss, test accuracy, f1-score, and balanced accuracy compared to training from scratch. With transfer learning, the model achieves a test accuracy of 98.20% and a balanced accuracy of 98.10%. Fine-tuning by unfreezing the last three blocks leads to a minor drop in accuracy metrics (0.14%) but substantially reduces computation time by 5.3 min. Further fine-tuning by unfreezing the last two blocks maintains similar performance, with a slightly larger drop in accuracy (0.55%), while achieving the lowest computation time, reducing it by 9.2 min, and highlighting an effective trade-off between accuracy and computational efficiency.

The DeiT-Base model, which consists of 12 blocks, demonstrates substantial performance improvements through transfer learning. This is evidenced by a decrease in test loss and notable improvement in accuracy and f1-score. Additionally, there is a slight reduction in computation time, showcasing the efficiency gains. When the last three blocks are unfrozen and fine-tuned, the model experiences a minor decrease in test accuracy by 1.55% but gains significant computational efficiency and a reduction in the number of parameters. Further efficiency is achieved by unfreezing the last two blocks, which results in even greater computational efficiency and parameter reduction, with only a slightly higher compromise in accuracy. The results of the DeiT-Base model highlight a clear and positive trade-off between accuracy and efficiency. Strategic fine-tuning by unfreezing specific blocks leads to significant gains in computational efficiency and parameter reduction, offering flexibility to optimize the balance between performance and efficiency according to specific application requirements.

**Figure 7:**
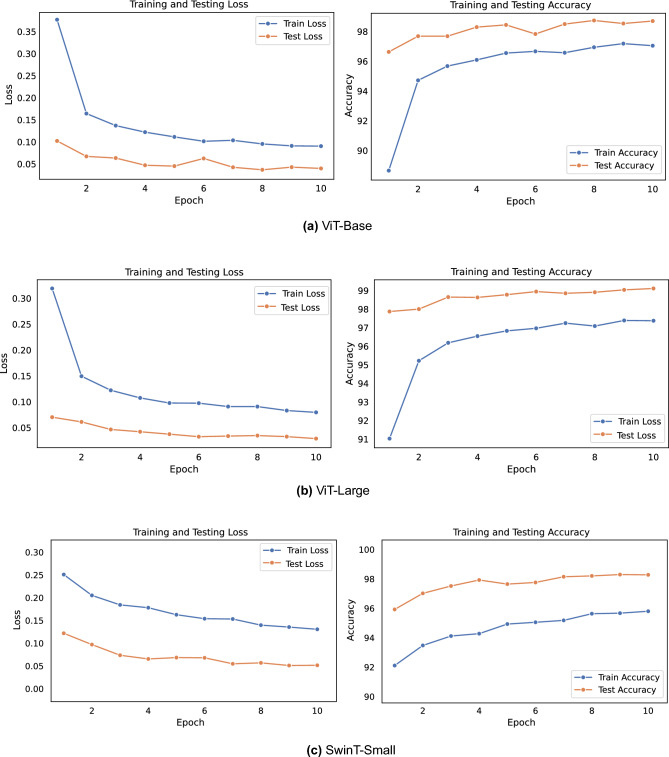
Accuracy and loss over vision transformer and SwinT-Small on the EuroSAT dataset.

**Figure 8:**
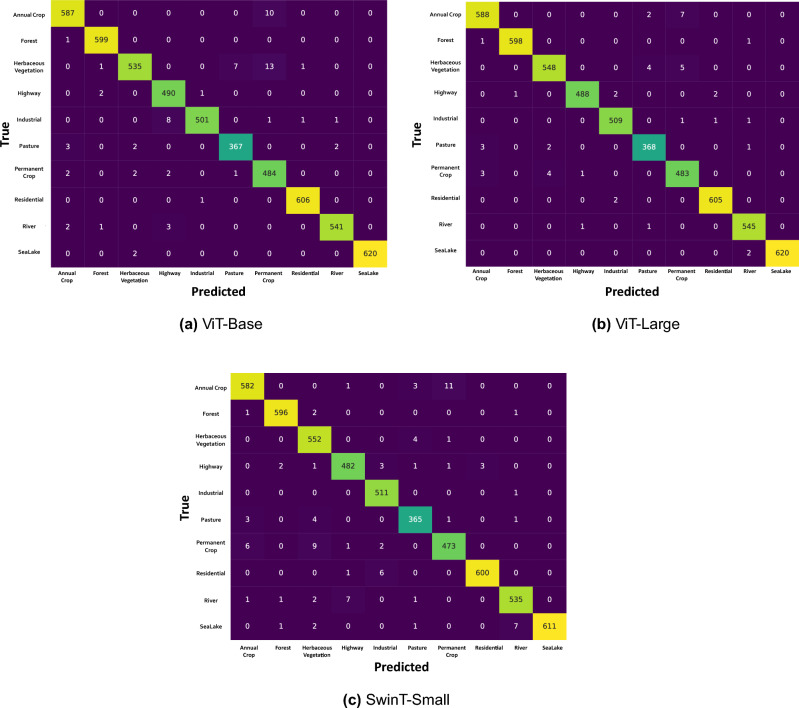
Confusion matrices showing true vs predicted labels across transformer-based models on the EuroSAT dataset.

**Figure 9:**
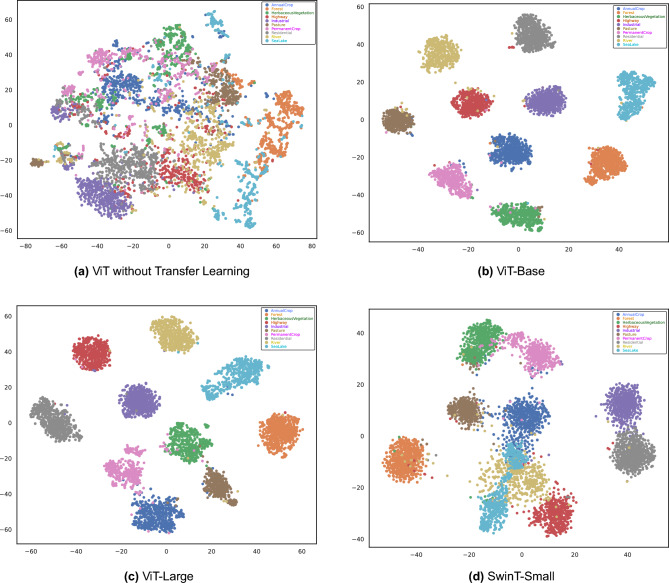
t-SNE visualization on EuroSAT dataset: distinctive patterns in vision transformer and swin transformer feature spaces.

The graphical analysis provided by Figs. 7, 8, and 9 complements the findings in Table 5. Figure 7 indicates the training progress of the ViT-Base and ViT-Large models. Both models exhibit a consistent decrease in loss and a steady increase in accuracy, indicating the effectiveness of their respective training processes. Although the SwinT-Small also displays a similar trend, its learning pattern differs, which may be due to its unique architecture. Figure 8 provides an in-depth look at the models’ predictive accuracy across different classes, with higher diagonal values indicating a strong true positive rate. Some misclassifications are evident, which presents a prospect for model refinement. In Fig. 9, we can see the t-SNE visualizations that demonstrate the effectiveness of transfer learning. Both the ViT-Base and ViT-Large models show well-defined clustering of features post-transfer learning, which leads to a clear separation between different classes. This visual representation aligns with the highly balanced accuracy presented in Table 5, particularly for the ViT-Large model, which has a balanced accuracy of over 99%.

Across all models, transfer learning stands out as a powerful method for enhancing performance across all metrics. Fine-tuning by unfreezing specific blocks appears to be a feasible strategy for reducing computation time and parameter count, albeit with a trade-off in terms of a slight drop in accuracy metrics. These results effectively showcase the balance that must be considered between performance, computational efficiency, and model size when training transformer-based architectures in LULC applications.

**Table 6:**
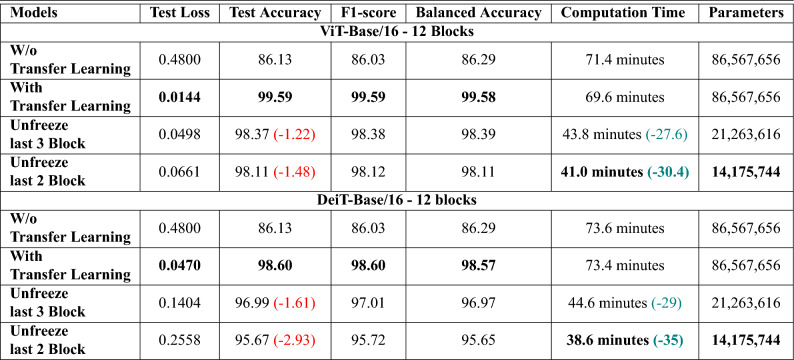
Performance comparison among transformer-based models utilizing transfer learning and fine-tuning on the EuroSAT dataset.

Significant values are in bold.

**Figure 10:**
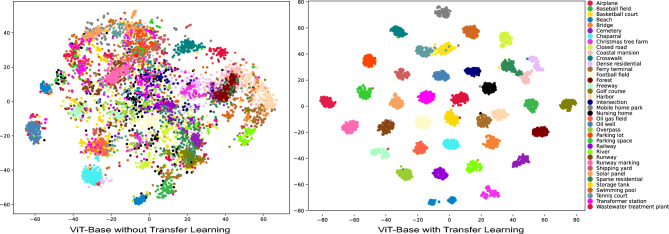
t-SNE Visualization demonstrating the performance of the vision transformer-base model before and after transfer learning on the PatternNet dataset.

#### Transfer learning and fine-tuning over transformer-based models on patternnet dataset

Evaluating high-resolution satellite imagery datasets can be challenging due to the limited availability of open-access resources. Many high-resolution datasets are proprietary and require paid subscriptions. Some open-access options are available, but they may have limitations in resolution and coverage. Therefore, we used the PatternNet^28^ dataset for further investigation.

Table 6 compares the performance of ViT-Base and DeiT-Base models using transfer learning and fine-tuning on the PatternNet dataset. Transfer learning has proven to be highly effective when applied to the ViT-Base model with 12 blocks, significantly enhancing the model’s performance. This approach results in test accuracy and f1-score of 99.59%, along with high Balanced Accuracy. When fine-tuning the model by unfreezing the last three blocks, there is a slight decrease in test accuracy to 98.37% and an increase in test loss to 0.0498. However, this decline is marginal and generally considered acceptable. The fine-tuning strategy offers substantial computational benefits, reducing computation time by almost 27.6 min and a significant decrease in model parameters.

Similarly, the DeiT-Base model shows improvements with transfer learning, achieving a test accuracy and f1-score of 98.60%. Unfreezing the last three blocks for fine-tuning results in a slight drop in test accuracy to 96.99% and an increase in test loss to 0.1404. However, computation time is reduced by 29 min, and there is a significant decrease in parameters. This efficiency gain is advantageous for practical applications with resource constraints.

Unfreezing only the last two blocks during fine-tuning further reduces computation time and model complexity. For the ViT-Base model, test accuracy decreases to 98.11% with a test loss of 0.0661, and computation time is reduced by 30.4 min, with parameters dropping to 14,175,744 from 86,567,656. For the DeiT-Base model, test accuracy declines to 95.67% with a test loss of 0.2558 and computation time is minimized to 38.6 min, with a decrease in model parameters. This suggests a diminishing return on efficiency gains with fewer blocks unfrozen but highlights the customizable nature of the fine-tuning process to balance model performance and computational efficiency.

Figure 10 illustrates the t-SNE visualizations of the ViT-Base model applied to the PatternNet dataset, comparing the results without transfer learning and with transfer learning. These visualizations underscore the effectiveness of transfer learning in enhancing the ViT-Base model’s ability to distinguish between different classes. The distinct clusters in the right panel reflect the model’s improved performance metrics, as seen in Table 6, including higher test accuracy, F1-score, and balanced accuracy. This improved feature separation is crucial for better classification results, particularly in complex datasets like PatternNet. The comparison between the two visualizations highlights the importance of transfer learning in transformer-based models for practical applications.

In summary, transfer learning significantly boosts the performance of both ViT-Base and DeiT-Base models on the PatternNet dataset. Fine-tuning by unfreezing the last few blocks provides a good trade-off between performance and computational efficiency, making it beneficial for applications where resource constraints are a major concern.

**Figure 11:**
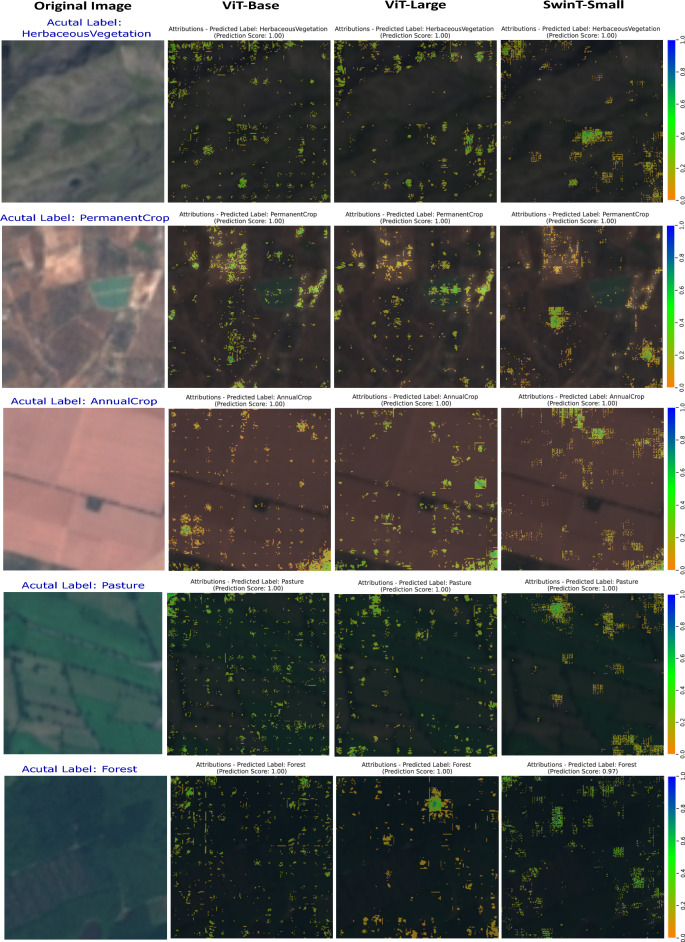
Attribution maps representing different LULC Classes across transformer-based models on the EuroSAT dataset.

### Model interpretation with explainable AI (XAI)

This section presents the results obtained from the LULC explainability block of our proposed framework. We first showcase the attribution maps generated from model predictions using the Captum library’s Integrated Gradients tool to highlight influential image regions. We then compare these results with other tools for further validation.

**Figure 12:**
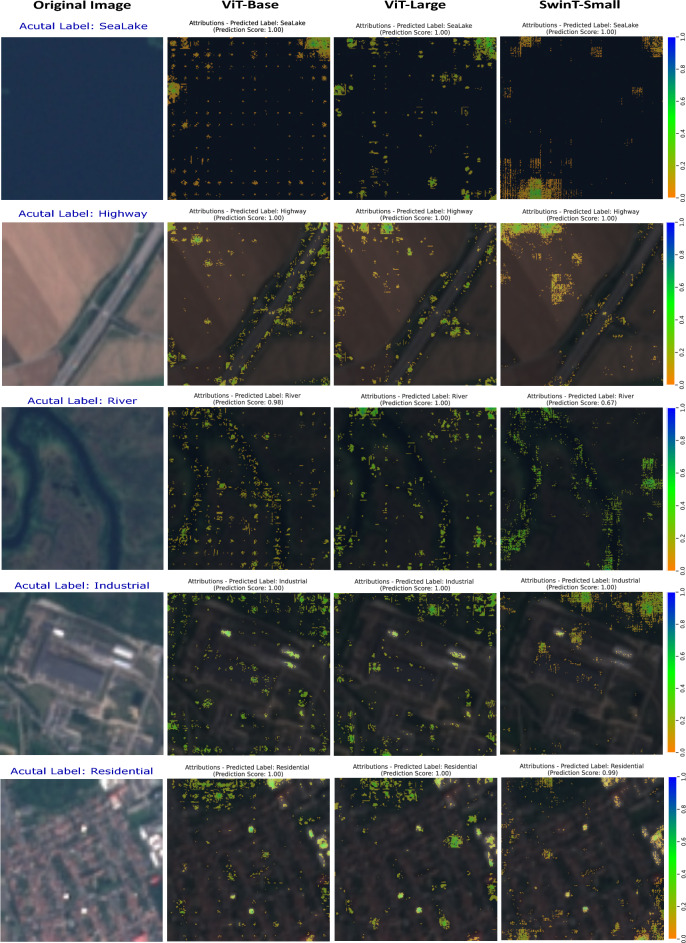
Attribution maps representing different LULC Classes across transformer-based models on the EuroSAT dataset.

Figures 11 and 12 showcase visualizations, contrasting with the original images derived from transformer-based architectures like ViT-Base, ViT-Large, and SwinT-Small. The resulting visual overlay, which ranges in shade from orange to blue, signifies the pixel-level impact on the model’s predictions. This intricate analysis, reinforced by prediction scores and a 95th percentile threshold for Integrated Gradients, highlights only the top 5% most important features. This thresholding approach ensures that the visualizations emphasize the most significant areas influencing the model’s decisions, providing a clear and focused understanding of the key features driving the predictions. This detailed insight is crucial for analyzing and enhancing the models’ capability to distinguish nuanced patterns and features in diverse LULC categories.

The *HerbaceousVegetation* classification in Fig. 11 illustrates the models’ proficiency in identifying specific features, with both ViT-Base and ViT-Large achieving 100% prediction scores. SwinT-Small also matches this accuracy, adept at identifying complex vegetation structures, demonstrating explainable AI’s usefulness in environmental study.

In the *PermanentCrop* class, also in Fig. 11, all models achieve a 100% score. ViT-Base focuses on specific crop attributes, while ViT-Large’s broader analysis differentiates PermanentCrop land from similar types. SwinT-Small highlights nuanced structural elements, showcasing the models’ capabilities in the differentiation of agricultural land types.

The analysis of the *AnnualCrop* category reveals each model’s ability to identify distinct agricultural patterns, all achieving a 100% score. This demonstrates their proficiency in recognizing complex patterns in AnnualCrop landscapes, which is essential for transparent and practical agricultural analysis.

In the analysis of the *Pasture* class, as shown in Fig. 11, all models demonstrate remarkable accuracy. The ViT-Base model with a 100% prediction score, focuses on specific pasture markers. ViT-Large, achieving a perfect score, captures more extensive environmental features, showcasing its ability to assess the pasture in its broader landscape context. Similarly, the SwinT-Small, with a 100% score, indicates a comprehensive evaluation of spatial distribution within the pasture. This precise classification by all models is crucial for effective land use and agricultural management, highlighting their reliability in environmental monitoring.

Turning to the *Forest* class in the same figure, the models exhibit their adeptness in identifying key forest characteristics. ViT-Base, achieving a 100% score, effectively pinpoints specific forest features like dense canopies and clearings. ViT-Large also reaches a 100% score, likely focusing on the expansive forest landscape and its integration within a larger ecosystem. The SwinT-Small, with a slightly lower score of 97%, appears to delve into more intricate forest details, possibly assessing complex edges or heterogeneous areas of the forest. These high-performance scores across the models showcase their nuanced and transparent approach to forest classification, underscoring the strengths of explainable AI in detailed environmental understanding.

The attribution maps for the *SeaLake* class, as shown in Fig. 12, demonstrate how each model determines the necessary pixels for accurate class classification. ViT-Base identifies discrete, scattered pixels, while ViT-Large recognizes more distributed patterns. Contrarily, SwinT-Small focuses on densely attributed regions, indicative of its sensitivity to structured elements like edges and textures specific to the SeaLake class. These distinct behaviors lead to correct predictions with a 100% score, and the maps visually communicate each model’s unique interpretative strategies, offering insights essential for environmental monitoring.

For the *Highway* class, depicted in the same figure, the attribution maps show the models’ decision-making criteria in classifying images with 100% precision. ViT-Base targets specific features like lane markings, ViT-Large offers a denser pixel pattern capturing broader structural and textural aspects, and SwinT-Small assesses the highway in its broader context and geography. These varied analytical angles are crucial for geographic and ecological assessments.

In the *River* class, Fig. 12 showcases ViT-Base with a 98% score, emphasizing specific river features, and offering detailed insight into the model’s focus. In contrast, ViT-Large, scoring 100%, presents a denser heatmap for a more holistic analysis. SwinT-Small, with a 67% score, considers the river’s broader spatial context, offering a unique perspective on feature relevance. These models transform complex inner workings into understandable visual forms, enhancing transformer model explainability in satellite imagery interpretation.

All the models in Fig. 12 under the *Industrial* category performed exceptionally well in identifying industrial characteristics with a prediction score of 100%. However, what distinguishes them apart from each other is the valuable information provided by the attribution maps. These maps reveal diverse patterns in pixel attribution, providing insights into the models’ ability to distinguish between various parts of industrial attributes. The transparency in the decision-making process proves the effectiveness of transformer-based models in detecting industrial patterns, contributing significantly to model explanation.

In the *Residential* category, ViT-Base focused on widespread influential zones, leading to a 100% prediction score. ViT-Large displays high attribution scores in larger clusters, while SwinT-Small, with a 99% score, focuses on compact, brightly highlighted salient features. Despite high confidence across all models, the attribution maps reveal fine distinctions in their interpretative strategies.

**Figure 13:**
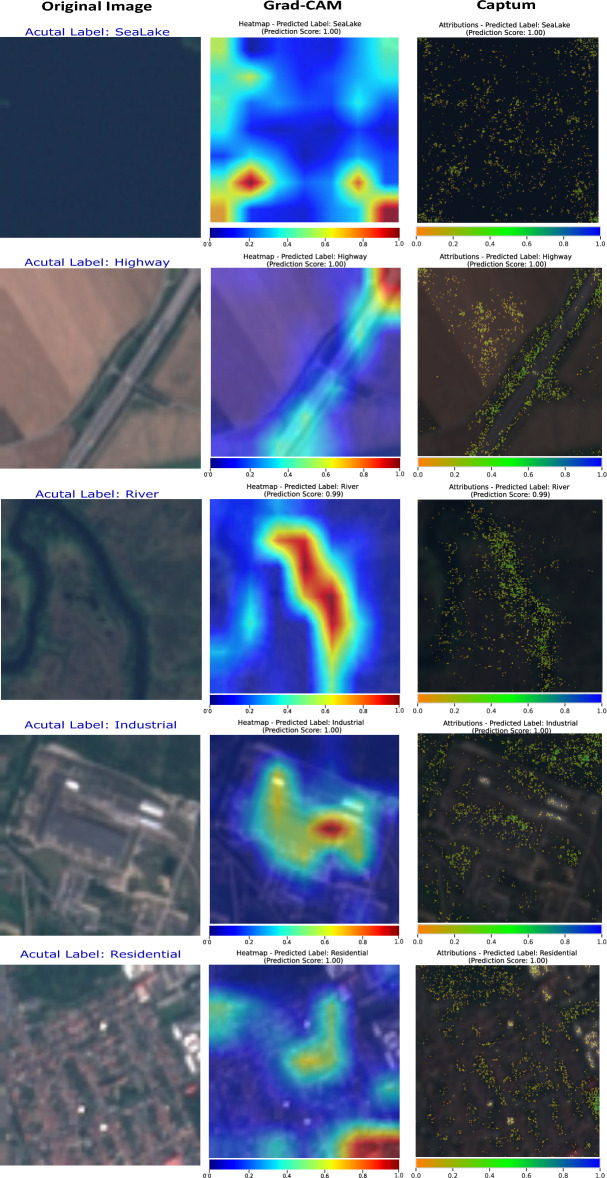
Comparison of Grad-CAM and captum on DenseNet161 model using the EuroSAT dataset.

#### Captum versus other method

In order to compare the performance of Captum with the state-of-the-art method, we selected Grad-CAM^19^. This approach allowed us to demonstrate the strengths and applications of both interpretability tools effectively. While Grad-CAM is a powerful tool for CNNs, it is not ideally suited for transformer-based models due to architectural differences, which are discussed in detail in Table 1. Therefore, we used DenseNet161^32^, an advanced convolutional neural network, to evaluate Captum and Grad-CAM for model interpretation in LULC analysis. Captum is a model-agnostic tool, making it versatile for various types of neural network architectures.

Figure 13 illustrates the performance of Grad-CAM and Captum using DenseNet161 model for LULC classification. Grad-CAM provides localized visual explanations by utilizing the gradients of the target class flowing into the final convolutional layer, generating heatmaps that highlight regions of the image most relevant to the model’s predictions. While effective for CNNs, Grad-CAM’s applicability is limited to models with convolutional layers. In contrast, Captum offers a model-agnostic approach that supports multiple interpretability techniques, with Integrated Gradients being particularly notable. Integrated Gradients attribute the prediction to each input feature by approximating the integral of gradients along the path from a baseline to the input, providing a detailed and comprehensive understanding of influential image regions. This method highlights subtle and nuanced features contributing to the model’s decision-making process, offering more profound insights into model behavior. Captum’s flexibility allows it to be used across various neural network architectures, including transformers, making it a versatile tool for comprehensive model interpretation. Figure 13 shows examples of the interpretability outputs for different LULC classes: SeaLake, Highway, River, Industrial, and Residential. Grad-CAM heatmaps and Captum attributions highlight the relevant regions in the original images that contribute to the model’s predictions. Specifically, Captum’s Integrated Gradients method is used with a 95th percentile threshold, meaning that only the top 5% most important features are highlighted in attribution maps. This thresholding approach ensures that the visualizations emphasize the most significant areas influencing the model’s decisions, providing a clear and focused understanding of the key features driving the predictions. In our comparison, Captum, through Integrated Gradients, demonstrated its ability to provide richer and more detailed visualizations, revealing the intricate features that influence model predictions. This detailed level of insight is crucial for understanding complex models and ensuring accurate and reliable LULC analysis. Consequently, while Grad-CAM remains a powerful tool for quick and intuitive visual assessments, Captum’s Integrated Gradients method stands out for its depth of analysis and versatility.

The results presented in this section highlight the pivotal role of our explainability block in ensuring fairness, transparency, and trust in AI-driven decisions, particularly in LULC applications. Attribution maps are crucial for improving computer vision tasks and interpreting satellite imagery in environmental surveillance and urban/rural planning. They promote ethical AI decisions and identify potential biases in model outputs, enhancing the effectiveness and practicality of transformer-based models across various fields. Beyond these primary benefits, attribution maps make AI more accessible, facilitating interdisciplinary collaboration essential for informed decision-making in climate change and sustainable development. These insights help refine AI models, promoting technological advancement and ethical, transparent solutions to global challenges. Additionally, our framework offers a model-agnostic solution to ensure that interpretations are consistent across different models and datasets, enhancing the robustness and reliability of our framework.

### Model generality and scalability

Our framework is rigorously tested on two distinct datasets to evaluate its generality and scalability, demonstrating its robustness and versatility in LULC classification tasks. The first dataset, EuroSAT, comprises Sentinel-2 imagery and provides detailed LULC classification from satellite images. This dataset allowed us to assess the framework’s accuracy in identifying and classifying various land cover types. The second dataset, PatternNet, contains high-resolution aerial imagery, offering a different perspective and helping us evaluate the model’s performance under varying conditions.

The EuroSAT dataset includes LULC classes such as annual crop, forest, herbaceous vegetation, highway, industrial, pasture, permanent crop, residential, river, and seaLake. This dataset tested the model’s precision in distinguishing fine details and subtle variations in land cover types. Conversely, the PatternNet dataset presented diverse challenges with classes like airplane, dense residential, intersection, parking lot, railway, river, runway, and sparse residential. The varying scales and levels of detail in this dataset were crucial for evaluating the model’s robustness and adaptability.

To provide a comprehensive evaluation, we incorporated different types of architectures from both CNN and transformer families in our study. This approach allowed us to balance traditional convolutional neural networks with modern transformer-based models, assessing their respective strengths and weaknesses in LULC classification.

Our experiments demonstrated that our framework effectively generalizes across diverse types of imagery, model architectures, and LULC classes. We employed multiple evaluation metrics, including accuracy, precision, recall, F1-score, processing time, and memory usage, to assess the model’s performance comprehensively. Additionally, we conducted an ablation study by replacing Captum with Grad-CAM and observed that the framework maintained comparable performance. The results indicate that our framework exhibits strong generalization capabilities across different datasets, models, and interpretability tools while maintaining high performance.

These findings suggest that our framework has significant potential for practical applications in remote sensing, environmental monitoring, and other fields requiring robust image classification and interpretation. We recognize that further research is needed to expand the scope of practical applications and investigate the generality and scalability of the framework on even larger-scale datasets and related tasks.

### Challenges in practical applications

Implementing transformer-based models for LULC classification is promising, but it comes with its set of challenges. Some of the practical challenges related to this work include:

- Acquiring high-resolution satellite imagery data is challenging due to the limited availability of archived data and the high costs associated with obtaining new data. Ensuring consistent and comprehensive datasets for effective model training and evaluation is essential.
- Deploying transformer-based models in real-world applications presents significant computational challenges because of their complexity and resource requirements. This paper proposes a framework that addresses these challenges by utilizing transfer learning and fine-tuning, all of which contribute to optimizing model deployment and making it more feasible for practical applications.
- To maintain the accuracy of a model over time, regular updates and retraining are necessary due to environmental changes and land use dynamics. These changes can significantly impact the performance of the model. Therefore, it is crucial to continuously improve the model to adapt to these changes and sustain its effectiveness.
- Understanding and interpreting the decisions made by transformer-based models can be difficult due to their complexity. Developing methods to make these models more transparent and interpretable is essential for helping stakeholders trust and effectively use the insights provided. Therefore, in this paper, we integrated Captum into our framework, which is model agnostic and offers advanced interpretability techniques for all kinds of models, including transformers. This integration enhances the transparency and interpretability of the models, allowing stakeholders to trust and effectively utilize the insights provided.

Addressing these practical challenges is crucial for the successful deployment of transformer-based models in real-world LULC applications and beyond.

## Conclusion

This study demonstrates the significant impact of transfer learning and fine-tuning on enhancing the efficiency and usability of transformer-based models, especially Vision Transformer and Swin Transformer variants in LULC classification. These strategies effectively balance computational cost and accuracy, thereby improving the models’ performance, which is crucial for various applications. Fine-tuning, particularly, has effectively reduced processing demands while maintaining high precision. Additionally, our framework integrates Captum to provide a model-agnostic approach that emphasizes the importance of model interpretability, ensuring transparency and trust in these AI models and offering critical insights for LULC data-driven decision-making processes. The primary contributions of this research lie in enhancing the practical application of transformer-based models in LULC analysis and promoting their ethical use by improving fairness and transparency. Our findings lay the groundwork for future advancements in computer vision and LULC, focusing on developing efficient, reliable, and comprehensible deep learning applications.

In the future, we plan to broaden the scope of our work to further explore its practical applications. We aim to investigate the generalizability and scalability of our framework on larger datasets and related computer vision downstream tasks. Additionally, we intend to focus on using a wider range of spectral bands in satellite imagery to enhance the reliability and interpretability of our framework in LULC research.

## Data Availability

The dataset used during the study is open-source and available online in the EuroSAT repository^27^ and PatternNet^28^. The code supporting the results of this study is available on GitHub https://github.com/Ci2Lab/Mehak_Transformer_LULC_XAI.
